# Wunderlich Syndrome Triggered by Deadlifting: A Rare Case Report of Spontaneous Renal Haemorrhage

**DOI:** 10.1155/criu/3951765

**Published:** 2025-12-05

**Authors:** Katie McComb, Samuel Bishara, Abhisekh Chatterjee, Konstantinos Charitopoulos, Ivo Donkov, Norma Gibbons, Nisha Pindoria, Panagiotis Nikolinakos

**Affiliations:** ^1^ Department of Urology, West Middlesex University Hospital, Chelsea & Westminster Hospital NHS Foundation Trust, London, UK, nhs.uk; ^2^ Imperial College School of Medicine, Imperial College London, London, UK, imperial.ac.uk; ^3^ Department of Urology, Imperial College Healthcare NHS Trust, London, UK, nhs.uk

**Keywords:** angiography, disseminated intravascular coagulation, interventional radiology, nephrectomy

## Abstract

**Background:**

Wunderlich syndrome (WS) is a rare phenomenon. It was first described by Carl Wunderlich in 1857 as a clinical picture of spontaneous renal bleeding with dissection of blood into either or both the subcapsular and perinephric spaces. Tumours, vascular malformations, coagulopathy and inflammatory states have been shown to be aetiological factors, yet up to 10% of cases remain idiopathic. WS is usually diagnosed on CT imaging due to the variable history and signs on examination which can mimic many other abdominal conditions. Management strategies include conservative treatment, embolisation and surgical nephrectomy.

**Case Presentation:**

A 37‐year‐old female presented to the emergency department with acute onset severe right flank pain after performing deadlift exercises in the gym. She collapsed on arrival. Clinical observations and blood results were indicative of haemorrhagic shock. Computed tomography (CT) with angiography demonstrated an acute right renal haemorrhage with active extravasation and the formation of a large perirenal haematoma. Blood products and vasopressors were administered to resuscitate the patient. Definitive management with embolisation was undertaken by the interventional radiology team.

**Conclusion:**

WS is a rare diagnosis that should be considered in patients presenting with flank pain and evidence of haemorrhagic shock. Weightlifting can act as a trigger for this condition. Early recognition and intervention can enable successful resuscitation and definitive treatment with minimally invasive embolisation.

## 1. Introduction

Spontaneous renal haemorrhage is rare [1–4]. Rather, renal haemorrhage is usually associated with trauma or procedures [5]. Wunderlich syndrome (WS) describes the occurrence of spontaneous renal haemorrhage [1–4]. It was first described by Carl Wunderlich in 1857 as a clinical picture of spontaneous renal bleeding with dissection of blood into either or both the subcapsular and perinephric spaces [6]. It describes intrarenal and perirenal haemorrhage in the absence of trauma or anticoagulation [1, 3, 4]. It is usually associated with tumours, vascular malformations, vasculitis and coagulopathic states [1, 7, 8]. WS is uncommon with a large meta‐analysis demonstrating only 165 cases over a 15‐year period from 1985 to 1999 [6]. Within this, only 11 cases were considered to be idiopathic and exercise was not identified to be a trigger [6]. WS can be life‐threatening, resulting in haemorrhagic shock and often requires emergency intervention [9].

WS is difficult to diagnose. Classically, it presents with Lenk′s triad of flank pain, a palpable flank mass and signs of hypovolaemic shock [1–3, 8]. However, the symptoms are variable and mimic other acute abdominal conditions [7]. Consequently, it is frequently diagnosed following imaging [4]. Historically, WS was managed with emergency surgical intervention and nephrectomy [3]. More recently, conservative management strategies and minimally invasive embolization techniques have been shown to be effective definitive management options of WS. We present an idiopathic case of WS, in a 37‐year‐old female patient, triggered by weightlifting. It was successfully managed with embolisation.

## 2. Case Presentation

A 37‐year‐old Asian female presented to the emergency department with right flank pain of approximately 1 h duration. Her past medical history included hypertension during pregnancy which had resolved postpartum. She had no other significant past medical or surgical history and did not take any regular medications. This pain occurred suddenly whilst performing a deadlift in the gym earlier in the day, causing her to drop the weight. The pain progressively worsened. On arrival in the emergency department, she was clinically shocked with evidence of tachycardia and hypotension. Her heart rate was 110 beats per minute with a blood pressure of 94/65 mmHg. She was noted to be tender in the right flank and lumbar region on examination. There was no palpable mass. A urinary catheter was inserted which drained clear urine. Repeated venous blood gas assessments demonstrated an acute haemoglobin drop from 122 to 101 g/dL within 2 h. A Focused Assessment with Ultrasonography for Trauma (FAST) scan did not demonstrate any free fluid intraperitoneally but the right kidney appeared to have abnormal morphology. A computed tomography (CT) scan with angiography, of the abdomen and pelvis, was performed. The patient became haemodynamically unstable after the scan with a heart rate of 70 beats per minute and an associated blood pressure of 87/50 mmHg despite intravenous fluids. A major haemorrhage protocol was initiated [10]. Three units of packed red cells, 2 g of intravenous tranexamic acid, 10 mL of 10% intravenous calcium gluconate, intravenous crystalloids and 1 unit of fresh frozen plasma were administered. A further drop in Hb to 84 g/dL within 1 h was detected as demonstrated in Table 1. Due to persistent hypotension, vasopressor support with ephedrine was administered by the anaesthetic team [11]. The CT was reported as “acute right renal injury with associated active haemorrhage and large volume retroperitoneal haematoma. Haemoperitoneum noted. Right pleural effusion with basal atelectasis.” Figure 1 demonstrates the large area of haemorrhage surrounding the right kidney.

**Table 1:** Table demonstrating the blood results taken from formal analyses. Haematological, biochemical and coagulation results are demonstrated.

**Haemoglobin**	**84 g/dL**
White cell count	17.1 × 10^9^/L
Platelet	273 × 10^9^/L
Prothrombin time	15.8 s
Activated partial thromboplastin time	31.6 s
Sodium	135 mmol/L
Potassium	2.9 mmol/L
Chloride	106 mmol/L
Urea	6.2 mmol/L
Creatinine	81 *μ*mol/L
Estimated glomerular filtration rate	80 mL/min/1.73 m^2^

Figure 1Coronal CT angiography with premesenteric phase (a), arterial phase (b) and late mesenteric phase (c). Red arrow demonstrating acute right renal injury with loss of cortex, active haemorrhage and haematoma formation around the right kidney.

**Figure:**
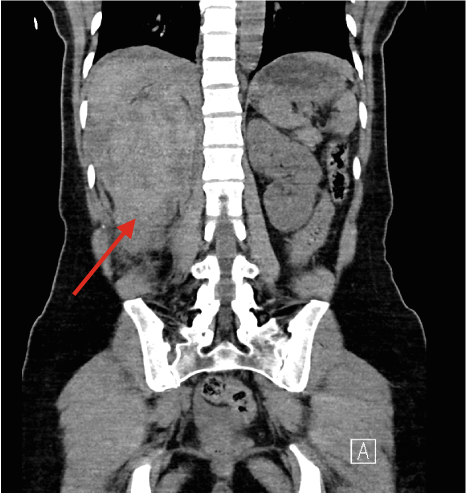
(a)

**Figure:**
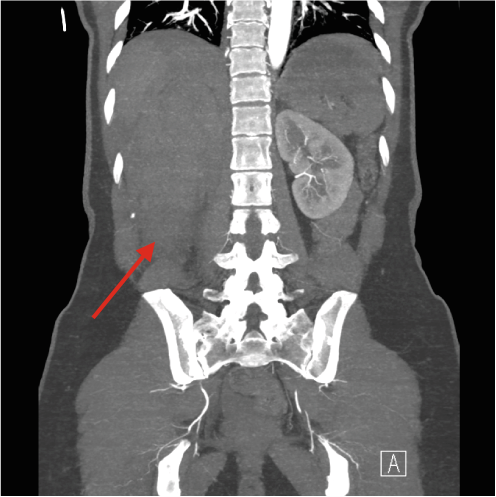
(b)

**Figure:**
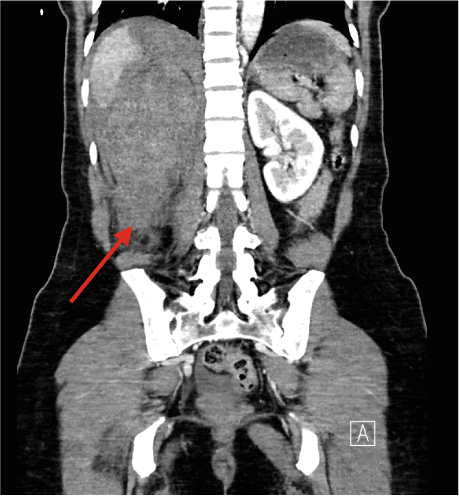
(c)

After resuscitation, the patient was transferred to the local trauma centre for embolisation by the interventional radiology team. A common femoral arterial puncture was performed to access the right renal artery. Active extravasation from two right inferior interlobar renal arteries was noted. Figure 8 coils were successfully deployed into these arteries, with no further active extravasation noted. Intravenous antibiotics were commenced, and strict bed rest was implemented. Subsequent CT triple phase imaging, shown in Figure 2, demonstrated a good result from the coil embolisation with no further extravasation, active bleeding or pseudoaneurysm formation.

Figure 2Axial CT imaging with arterial (a), portal venous (b) and delayed (c) phases demonstrating loss of right renal cortex and surrounding right perinephric haematoma without extravasation. Red arrows denote the position of the Figure 8 coils.

**Figure:**
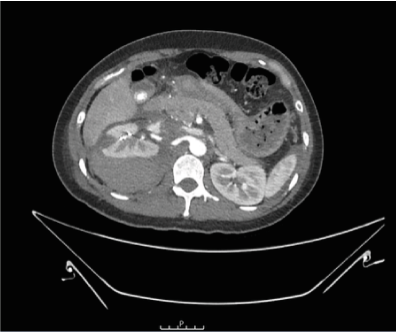
(a)

**Figure:**
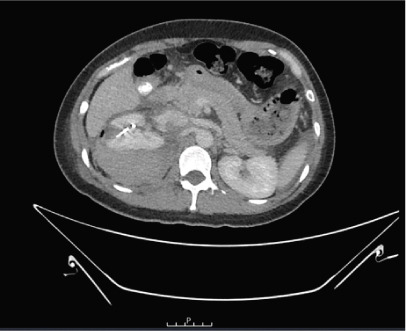
(b)

**Figure:**
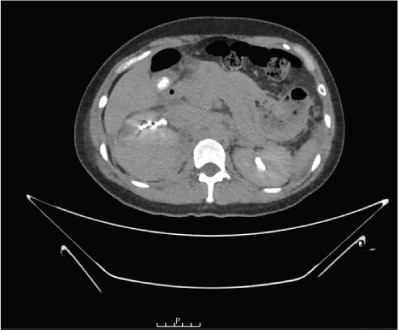
(c)

**Figure 3:**
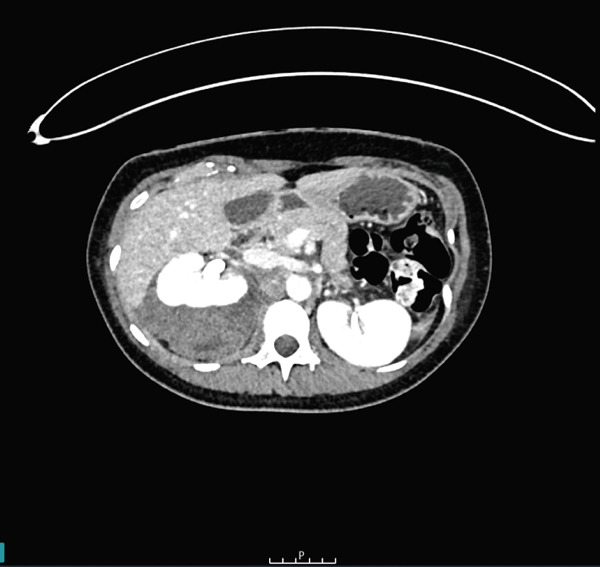
Axial, contrast‐enhanced CT image with urographic phase demonstrating persistent right perinephric haematoma without evidence of contrast extravasation from the pelvicalyceal system.

The large perinephric haematoma was seen to have a compressive effect on the renal veins bilaterally and on the inferior vena cava without thrombus formation. Further CT imaging with a urographic phase was performed 4 days later as shown in Figure 3. The appearances of the haematoma and its associated venous compression were stable. A calyceal or urinary leak was not seen. There was no evidence of underlying causative factors such as tumour or vascular malformation.

**Figure 4:**
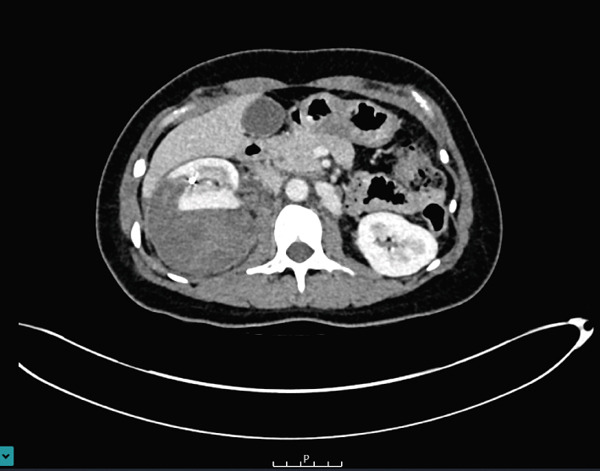
Axial CT image demonstrated a stable in size perinephric haematoma extending to the right psoas muscle.

During her admission, she was found to be hypertensive and blood pressure management was recommended on discharge. Follow‐up imaging, 1 week after discharge, demonstrated that the perinephric haematoma remained stable in size at 24.5 cm extending to the right psoas muscle as shown in Figure 4.

## 3. Discussion

WS is a serious and life‐threatening condition [1]. It is rare with only 102 cases reported in English literature between 2000 and 2016 [12]. Classically, it presents with Lenk′s triad of flank pain, a palpable flank mass and signs of hypovolaemic shock [1–3, 8]. However, this triad is seen in less than a quarter of patients [7]. Flank pain is the most common presenting symptom, as demonstrated in this case, and is noted in more than two‐thirds of patients with WS [1, 4, 7]. Haematuria, commonly microscopic, is reported by two‐fifths of patients at presentation [1, 4]. As shown, WS is a challenging clinical diagnosis due to its rarity and the association of its presenting symptoms with other more common differential diagnoses including renal calculi and aortic dissection [3, 7, 13]. As demonstrated, patients can present in extremis with haemodynamic instability. Consequently, the diagnosis of WS is frequently made on review of imaging findings [2, 3, 7].

The reported underlying aetiologies of WS are renal neoplasms, vascular abnormalities and infection [1, 7, 8]. An association with renal angiomyolipomas and renal cell carcinomas is seen in approximately 60% of WS cases [1, 7, 14]. In this case, there was no associated history of malignancy. Vascular abnormalities account for up to 30% of cases and typically include aneurysms, pseudoaneurysm and thrombosis [1, 13]. More rarely, vasculitides and coagulopathies are causative factors [1, 12, 13]. Despite this, WS is frequently found to be idiopathic in up to 10% of cases [1, 14]. Considering the aetiology more generally, cardiovascular risk factors including diabetes and hypertension may predispose patients to the development of WS [1, 8]. Consequently, in this case, the patient′s untreated hypertension may have contributed to her development of WS.

Whilst the pathophysiology of WS is poorly understood, tumour factors such as vessel obstruction, damage and necrosis have been identified [1]. Comparatively, altered renal vein pressure has also been proposed as the underlying mechanism of injury [1]. Alternatively, we hypothesise that the raised intra‐abdominal pressure from weightlifting, on a background of untreated preexisting hypertension, may have resulted in the renal vessels being unable to withstand the patient′s increased blood pressure leading to haemorrhage. Chronic hypertension has been recognised to cause nephroangiosclerosis which results in renal artery thickening and hardening [15]. This results in the vessel′s inability to withstand the increased blood pressure [15].

Imaging is key in WS diagnosis. CT imaging including angiography demonstrates the diagnosis and defines the patient′s underlying renal anatomy [6, 9, 16]. It also assesses the extent of bleeding and identifies areas of active bleeding [6, 9, 16]. The sensitivity of CT imaging in the detection of perirenal haemorrhage is 100% [8, 14]. Acute haemorrhage is noted to be hyperdense to the renal parenchyma with an associated density of 40–70 Hounsfield units [4]. As explained in this case, imaging guides decision‐making, further management and intervention [1, 2]. It can also identify the underlying aetiology of the haemorrhage [1]. In addition, this case highlights the role of ultrasound in the diagnosis of intra‐abdominal haemorrhage in unstable patients. However, further investigation with CT is necessary to define and characterise the injury [3]. Other cases in the literature have also proposed that magnetic resonance imaging is useful when CT fails to identify the source of bleeding [1, 14].

Various management strategies are discussed in the literature. This includes conservative management for haemodynamically stable patients with rehydration, blood product replacement and pain management [2, 3, 7, 8]. However, conservative management strategies have been shown to result in reduced overall renal function compared to other treatments [2, 17].

Comparatively, intervention with surgical exploration and nephrectomy, has been proposed to be the first‐line treatment for unstable patients [2, 3]. However, embolisation, through interventional radiological approaches, has also been identified to be an effective alternative definitive management strategy [2, 3, 7]. This method, as portrayed in this case, is effective in controlling bleeding and has a lower risk profile for patients [2]. It is minimally invasive and results in nephron preservation which leads to improved overall kidney function compared to nephrectomy and conservative management [14]. As shown, it results in a short hospital stay for patients as well as a reduced risk of mortality [14]. Consequently, we propose that primary embolisation should be considered in cases of WS and nephrectomy should be reserved for cases of refractory bleeding or where embolisation is not readily available [9]. This case demonstrates the success of early intervention with embolisation, avoiding the need for surgery.

As portrayed, the prognosis of WS is good following early intervention to control bleeding in haemodynamically unstable patients. However, the consequences of WS can be significant. The extrinsic compression resulting from the haematoma or perirenal urinoma causes inappropriate activation of the renin–angiotensin–aldosterone system. This in turn causes systemic hypertension, known as a Page kidney [1]. On discharge, careful outpatient follow‐up is required to monitor blood pressure and renal function.

## 4. Conclusion

WS is a rare condition that can be caused by strenuous physical exertion such as deadlifting. Whilst it classically presents with Lenk′s triad, diagnosis can be challenging and should be considered in patients presenting with severe abdominal pain and haemodynamic instability. Early assessment with CT imaging is required to characterise the condition and assess for the underlying aetiology. Early intervention with embolisation or surgical nephrectomy is needed for unstable patients. We have demonstrated that embolisation is a successful, minimally invasive and definitive treatment in the management of WS.

## Conflicts of Interest

The authors declare no conflicts of interest.

## Funding

No funding was received for this research.

## Data Availability

No public dataset was used in the creation of this manuscript. Data sharing is not applicable to this article as no new data were created or analysed in this study.
